# The transcription factor ATOH8 is regulated by erythropoietic activity and regulates *HAMP* transcription and cellular pSMAD1,5,8 levels

**DOI:** 10.1111/bjh.12649

**Published:** 2013-11-16

**Authors:** Neeta Patel, Joe Varghese, Patarabutr Masaratana, Gladys O Latunde-Dada, Molly Jacob, Robert J Simpson, Andrew T McKie

**Affiliations:** 1 Division of Diabetes and Nutritional Sciences, Kings College LondonLondon, UK; 2 Department of Biochemistry, Christian Medical CollegeBagayam, Vellore, India; 3 Department of Biochemistry, Faculty of Medicine Siriraj HospitalBangkok, Thailand

**Keywords:** ATOH8, *HAMP*, iron, erythropoiesis, pSMAD1,5,8

## Abstract

ATOH8 has previously been shown to be an iron-regulated transcription factor, however its role in iron metabolism is not known. *ATOH8* expression in HEK293 cells resulted in increased endogenous *HAMP* mRNA levels as well as *HAMP* promoter activity. Mutation of the E-box or SMAD response elements within the *HAMP* promoter significantly reduced the effects of ATOH8, indicating that ATOH8 activates *HAMP* transcription directly as well as through bone morphogenic protein (BMP) signalling. In support of the former, Chromatin immunoprecipitation assays provided evidence that ATOH8 binds to E-box regions within the *HAMP* promoter while the latter was supported by the finding that *ATOH8* expression in HEK293 cells led to increased phosphorylated SMAD1,5,8 levels. Liver *Atoh8* levels were reduced in mice under conditions associated with increased erythropoietic activity such as hypoxia, haemolytic anaemia, hypotransferrinaemia and erythropoietin treatment and increased by inhibitors of erythropoiesis. Hepatic *Atoh8*mRNA levels increased in mice treated with holo transferrin, suggesting that *Atoh8* responds to changes in plasma iron. ATOH8 is therefore a novel transcriptional regulator of *HAMP*, which is responsive to changes in plasma iron and erythroid activity and could explain how changes in erythroid activity lead to regulation of *HAMP*.

Erythropoiesis is essential for life and is by far the body's largest user of iron, consuming almost two-thirds of the body's total iron. Increased erythropoietic activity has a rapid and dramatic effect on iron metabolism, which has been well documented (Finch, 1994). Increased erythropoietic activity generally results in an increase in the reticulocyte fraction in the blood with a concomitant fall in plasma iron as iron is used up by the developing erythrocytes. Increased erythropoietic activity is a powerful suppressor of the iron hormone hepcidin (*HAMP*) levels thereby allowing for more iron to be made available for erythropoiesis through increased intestinal iron absorption and iron release from macrophages via regulation of the hepcidin–ferroportin axis (Nicolas *et al*, 2002a; Weinstein *et al*, 2002). The suppression of *HAMP* by increased erythropoietic drive is not well understood and occurs even in conditions where liver iron levels are high and which would normally lead to increased *HAMP* levels, such as in β-thalassaemia (Nemeth & Ganz, 2006) and hypotransferrinaemia (Bartnikas *et al*, 2010). Thus the erythropoietic regulator appears to be capable of overiding the iron stores regulator of hepcidin.

Hepatic *HAMP* mRNA levels are regulated by three major stimuli: (i) tissue and serum iron concentration; (ii) inflammatory signals and (iii) erythropoieitic activity. Regulation of *HAMP* appears to occur mainly at the transcriptional level via various response elements within the *HAMP* promoter, such as the bone morphogenic proteins response elements (BMP-REs), signal transducer and activation of transcription 3 (STAT-3), cAMP response element binding protein (CREB), hepatocyte nuclear factor 4 (HNF4) and enhancer boxes (E-boxes) binding elements (Courselaud *et al*, 2002; Bayele *et al*, 2006; Wrighting & Andrews, 2006). The bone morphogenic protein (BMP) pathway is involved in regulating the responses of *HAMP* to changes in tissue iron via changes in hepatic BMP6 levels (Meynard *et al*, 2009; Ramos *et al*, 2011) and inflammatory signals act via STAT-3 resulting in activation of *HAMP* (Wrighting & Andrews, 2006).

Less is known about how changes in erythropoietic activity lead to altered hepatic *HAMP* levels, for example, what signals are sensed by the liver as a result of changes in erythropoiesis as well as the nature of the signal transduction mechanism are unclear. Both changes in serum iron and/or release of soluble factors from developing erythrocytes have been evoked as potential indicators of altered erythropoietic activity. The level of plasma holo-transferrin (transferrin saturation) changes rapidly as a result of altered erythropoietic activity and is a key modulator of liver *HAMP* levels (Bartnikas *et al*, 2010; Li *et al*, 2010) and thus could be one signal, however the signal transduction pathway leading to *HAMP* regulation has not been defined. On the other hand, soluble factors, such as growth differentiation factor 15 (GDF15) or twisted gastrulation factor 1 (TWSG1), produced by erythroid precursors, and bone morphogenic protein binding endothelial cell precursor-derived regulator (BMPER), produced by endothelial cells, have all been postulated to play a role in the suppression of *HAMP* (Tanno *et al*, 2007, 2009; Patel *et al*, 2012) by, in most cases, inhibiting BMP signalling. However, the roles of these molecules in the regulation of *HAMP* in other forms of anaemia and conditions with altered erythropoiesis have not been shown. Thus the molecular basis of erythropoietic regulation of *HAMP* remains unclear.

*Atoh8* (or *Math6*) was originally identified as a distant mammalian homologue of the drosophila neural gene Atonal (Inoue *et al*, 2001). The *Atoh8* mRNA encodes a basic helix loop helix (bHLH) transcription factor and is ubiquitiously expressed in mouse at least in embryonic tissues (Lynn *et al*, 2008). *Atoh8* has been implicated in development of various tissues although its physiological function remains unknown (Lynn *et al*, 2008; Yao *et al*, 2010). Early studies had suggested that knock-out of *Atoh8* was embryonic lethal in mice (Lynn *et al*, 2008) however, using an alternative targeting strategy, recent work has shown that mice survive *Atoh8* ablation with no obvious phenotype (Rawnsley *et al*, 2013). bHLH or E-box proteins, such as ATOH8, bind to a palindromic (canonical) core consensus DNA sequence 5′-CANNTG-3′ known as an E-box element, where NN is usually CG or TG (Blackwell *et al*, 1993). Two canonical E-boxes have been described within the core human *HAMP* promoter with the sequence 5′-CACGTG-3′ and have been shown to bind other bHLH factors, USF1 and 2, as well as MYC and MAX (Bayele *et al*, 2006). *Atoh8* was first linked with iron metabolism by Kautz *et al* (2008) who found that hepatic *Atoh8* mRNA levels were up-regulated in mice chronically fed a high iron diet and down-regulated in those fed an iron-deficient diet. Thus *Atoh8* appears to be the only known iron-regulated bHLH transcription factor. In addition we noted that *Atoh8* mRNA was strongly down-regulated in liver expression microarrays of Tfr^hpx/hpx^ mice (Patel *et al*, 2012), a mutant with a very high degree of liver iron overload, chronic anaemia and very low *Hamp1* levels. Given that the regulation of *Atoh8* was similar to *Hamp1* we hypothesized that ATOH8 may be a transcriptional regulator of *HAMP*.

Here we report that ATOH8 can activate *HAMP* transcription and regulate cellular levels of pSMAD1,5,8. Moreover, *Atoh8* mRNA and protein levels were regulated in mouse liver under various conditions with altered erythropoietic activity, providing a mechanistic link between erythropoiesis and *HAMP* transcription.

## Materials and methods

### Animals

Hypotransferrinaemic mice (HPX or Trf^hpx/hpx^) were bred and maintained as previously described (Simpson *et al*, 1991). Normal littermates (mixture of Trf^+/+^ and Trf^hpx/+^) were used as controls. Hypoxia was induced by placing 7-week-old male CD1 mice in a hypobaric chamber for 24–72 h, as previously described (Raja *et al*, 1989); controls of the same gender and age were maintained under normoxic conditions. Hamp1^−/−^ mice and wild type (WT) littermates (all female C57BL/6/129 mixed background, aged 5–7 weeks old) were injected intraperitoneally with 60 mg/kg body weight of neutralized phenylhydrazine (PHZ) or saline solution twice on consecutive days as previously described (Masaratana *et al*, 2011) and sacrificed 3 d after the last injection. For erythropoietin (EPO), Carboplatin, Apo and Holo transferrin treatments, male 6-week-old C57BL/6 mice were switched to a diet containing <4 ppm iron (TD.80396; Harlan Teklad, Madison, WI, USA) for 10 d to reduce the effect of the high iron chow diet on *HAMP* expression as previously described (Pak *et al*, 2006). Mice received intraperitoneal injection of either 200 units of EPO (Jansen Cilag Ltd, High Wycombe, UK), 2·5 mg of carboplatin or 200 units of EPO with 2·5 mg of carboplatin (Sigma-Aldrich, Gillingham, UK) dissolved in 100 μl saline on three consecutive days. Control mice received 100 μl of saline. Mice were sacrificed 24 h after the last injection. Apo and Holo transferrin (10 mg) dissolved in 100 μl of saline was injected i.p (control mice received saline alone). Mice were sacrificed 6 h later. Serum iron was measured with a liquid ferrozine-based Fe reagent (Thermo Electron, Melbourne, Vic., Australia). Tissue non-haem iron was determined as previously described (Masaratana *et al*, 2012). All animal experiments were performed under the authority of a UK Home Office license.

### Cell culture and *HAMP* promoter assays

HEK-293 cells were obtained from the American type culture collection (ATCC, Teddington, UK) and cultured in Dulbecco's modified Eagle's medium (DMEM; Sigma-Aldrich, Gillingham, UK) and 10% heat-inactivated fetal bovine serum (FBS; Sigma-Aldrich), penicillin-streptomycin and glutamine (Sigma-Aldrich). Cell cultures were maintained at 37°C under 95% air/5% CO_2_. Promoter assays employed approximately 0·9 kb of the human *HAMP* promoter (WT) cloned in the pGL3-basic luciferase reporter vector (Promega, Southampton, UK). E-box and BMP-RE mutated versions of this vector were kindly provided by Dr Pavle Matak (Department of Pharmacology and Cancer Biology, Duke University Medical Center, Durham NC, details can be found in Table SI). Reporter constructs were co-transfected into cells with a TK-renilla (3:1 ratio) using Fugene-6 (Roche Diagnostics, Burgess Hill, UK). Human ATOH8 –DDK (Flag) tagged plasmid (Origene technologies, Rockville, MD, USA) was co-transfected along with the reporter plasmids. Luminescence was detected using Dual-Luciferase Reporter Assay system and measured by luminometer (Promega).

### Western blotting and immunohistochemistry

Whole cell lysates were extracted from mouse liver or cultured HEK 293 cells by homogenization in 500 μl of radioimmunoprecipitation assay buffer (10 mmol/l Tris, 150 mmol/l NaCl, 1 mmol/l EDTA, 1% Nonidet P-40, 0·1% sodium dodecyl sulphate [SDS]) and protease inhibitor cocktail (1:200 dilution; Sigma Aldrich). The homogenates were centrifuged at 1000 × ***g*** at 4°C for 5 min. Nuclear protein from cells and tissues was extracted using the NE-PER nuclear and cytoplasmic extraction kit (Thermo Fisher Scientific, Loughborough, UK) according to the manufacturer's instructions. Protein was quantified using a protein assay (BioRad, Hemel Hempstead, UK) and resolved using pre-cast 10-12% reducing SDS polyacrylamide gel electrophoresis (SDS-PAGE; BioRad) before transfer to polyvinylidene difluoride (PVDF) membrane using a Trans blot Turbo (BioRad). Anti-ATOH8 and anti-DDK (FLAG) (Origene technologies) and pSMAD 1,5,8 (Cell Signaling Technology, Danvers, MA, USA) were used to detect the respective proteins. SMAD1 (Santa Cruz Biotechnology, Heidelberg, Germany) or beta actin (Sigma-Aldrich) were used as controls for protein loading. Blots were visualized by chemiluminescence (Thermo Fisher Scientific, Loughborough, UK). Immunohistochemistry was performed on cryostat sections of mouse liver as previously described (Patel *et al*, 2012) using anti-ATOH8 and fluorescein isothicyanate- conjugated secondary (Dako, Ely, UK). Sections were counterstained with propidium iodide (Vector Laboratories, Peterborough, UK) and images captured using Leica LS-2 confocal microscope (Leica Microsytems, Milton Keynes, UK).

### Quantitative polymerase chain reaction (qPCR)

**Figure 1:**
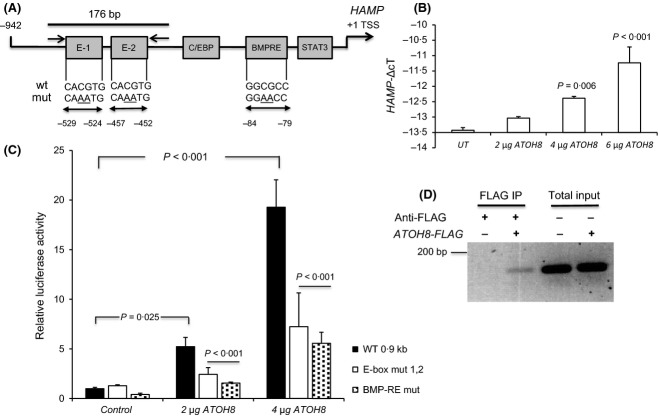
ATOH8 regulates *HAMP* transcription. (A) Schematic showing *HAMP* promoter and locations of bone morphogenic proteins response element (BMP-RE) and E-box region and mutations introduced. (B) Quantitative polymerase chain reaction (qPCR) assay of endogenous *HAMP* levels (normalized to *RPL19* plotted as –ΔcT values) in HEK 293 cells after transfection with 2, 4 or 6 μg ATOH8-FLAG plasmid. (C) *HAMP* promoter luciferase reporter assays in HEK 293 cells transfected with wild type (WT) *HAMP*, E-box mutant (E-box mut 1,2) or BMP-RE mutant (BMP-RE mut) after co-transfection with 2 or 4 μg of *ATOH8-FLAG*. Promoter activity was expressed relative to WT promoter activity without *ATOH8* co-transfection. Luciferase assays shown are means ± SD derived from a single experiment with three biological replicates and experiment shown is representative of at least three similar experiments. (D) CHIP assay: chromatin DNA was immunoprecipitated from untransfected HEK 293 cells or cells transfected with *ATOH8-FLAG*. Immunoprecipitation (IP) was performed using Anti-FLAG antibody. PCR (40 cycles) was performed on the IP material and 10% of the total input using primers flanking the E-box region (Fig 1A) and products run on a 1·5% agarose gel stained with ethidium bromide. Statistical analysis was performed using 1 or 2-way anova with Tukey's *post hoc* test.

One microgram of total liver RNA was reverse transcribed using a Transcriptor High Fidelity cDNA kit (Roche Diagnostics). All primers were designed using Universal Probe Library system (Roche Diagnostics) and qPCR was performed using an ABI PRISIM 7900 HT PCR machine (Applied Biosystems, Paisley, UK). Results were normalized to the housekeeping RNA *Rpl19*. Fold change was calculated using the method of Livak and Schmittgen (2001). In the case of *HAMP*, qPCR (Fig1B) results were normalized to the housekeeping RNA *RPL19* expressed as the negative of ΔcT. Details of primer sequences used are presented in Table SI.

### Chromatin immunoprecipitation (CHIP) assays

Chromatin immunoprecipitation assays were performed using a commercially available kit (Thermo Fisher Scientific). Chromatin DNA was prepared from untransfected HEK 293 cells and cells transfected with ATOH8-FLAG following the manufacturer's protocol and immunoprecipitated using Anti-FLAG antibody (Origene technologies, Rockville, MD, USA). PCR (40 cycles) was performed using the primers (5′ CCAGTTACCAGAGCCACATC 3′ and 5′ CAGGAGTGTCTGCATGTTG 3′), generating a 176 bp fragment encompassing the E-box 1 and 2 region (Fig1A). Control PCRs were performed using 10% of the input DNA.

### Statistical analysis

Data are presented as means ± SD. Statistical differences were determined using spss (IBM, Portsmouth, UK) where appropriate using either 1-way analysis of variance (anova) followed by Tukey's *post hoc* test or two-tailed Students *t*-test. 2-way analysis of variance (2-way anova) was used to test for significance between two or more groups and Bonferroni post-hoc test for interactions. A *P* value of <0·05 was considered as significant.

## Results

### ATOH8 regulates *HAMP* transcription and pSMAD1,5,8 levels *in vitro*

To test whether ATOH8 could play a role in regulating *HAMP* transcription, HEK-293 cells were transfected with increasing amounts of an *ATOH8-FLAG* tagged expression plasmid. Endogenous *HAMP* mRNA levels were increased by up to fourfold (*P* < 0·006) following transfection with increasing amounts of *ATOH8-FLAG* (Fig1B). In accord with this, *HAMP* promoter activity was increased by around 20- fold (*P* < 0·001) in cells transfected with *ATOH8* (Fig1C) and 0·9-kb of the human *HAMP* promoter fused to the luciferase gene (Fig1A). In both cases the effect of *ATOH8* was dose-dependent.

We next investigated the effect of mutation of the two E-box elements within the *HAMP* promoter previously shown to bind other bHLH proteins (Bayele *et al*, 2006). Mutation of the internal dinucleotide within the E-box elements from 5′-CACGTG-3′ to 5′-CAAATG-3′ (Fig1A) abolishes nuclear factor binding(Chen *et al*, 2012). Mutation of both E-boxes attenuated ATOH8-dependent *HAMP* promoter activity by more than 50% (*P* < 0·001) when compared to the WT promoter treated with ATOH8 (Fig1C). In addition, mutation of the BMP response element (BMP-RE) resulted in a 50% (*P* < 0·001) reduction in ATOH8 dependent *HAMP* promoter activation compared to WT treated with ATOH8 (Fig1C). To provide evidence for promoter occupancy by ATOH8 we performed CHIP assays using HEK 293 cells transfected with *ATOH8-FLAG*. Using the Anti-FLAG antibody as the immunoprecipitation (IP) antibody we were able to amplify a 176 bp band encompassing the E-box 1 and 2 regions of the *HAMP* promoter from cells transfected with *ATOH8-FLAG* but not untransfected cells (Fig1D).

**Figure 2:**
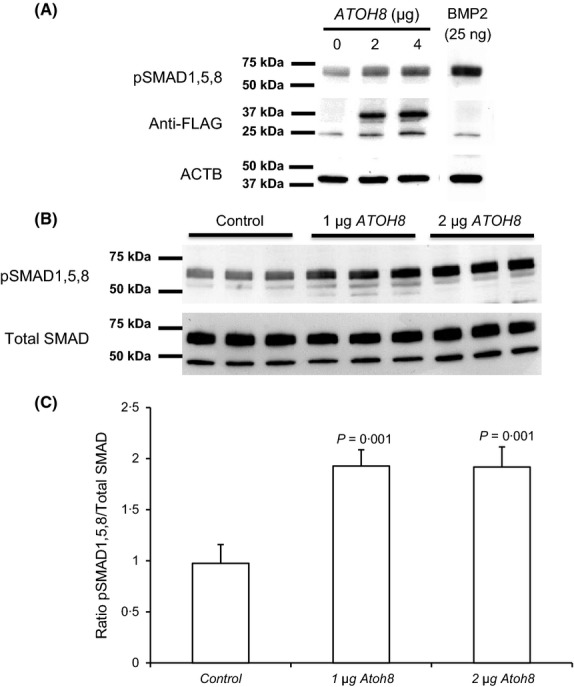
ATOH8 transfection increases pSMAD1,5,8 levels. (A) Western blot showing pSMAD 1,5,8 levels in HEK 293 cells after transfection with increasing amounts of *ATOH8-FLAG* (0, 2 and 4 μg plasmid DNA) compared with 25 ng of BMP2 as a positive control; lower panels show same blot re-probed with Anti-FLAG and ACTB (β-actin) antibodies. All lanes in A were run on the same gel, blotted and processed together and are from the same exposure. (B) Western blot of pSMAD 1,5,8 levels in HEK cells after transfection with *ATOH8* in comparison with total SMAD. (C) Densitometry of Western blots in B showing ratio of pSMAD1,5,8 to total SMAD. Statistical analysis was performed using 1-way anova with Tukey's *post hoc* test.

We next investigated whether *ATOH8* transfection affected pSMAD 1,5,8 levels in HEK 293 cells. *ATOH8* transfection significantly increased pSMAD 1,5,8 levels (*P* = 0·001, Fig2C) in a dose-dependent fashion (Fig2A,B). Hence, ATOH8 appears to regulate *HAMP* in two ways, firstly by acting directly on the *HAMP* promoter via E-boxes and indirectly through increased pSMAD1,5,8 levels. This may explain why mutation of E-boxes does not fully repress *HAMP* promoter activity.

### Regulation of hepatic ATOH8 levels in mouse models with altered erythropoietic activity

**Figure 3:**
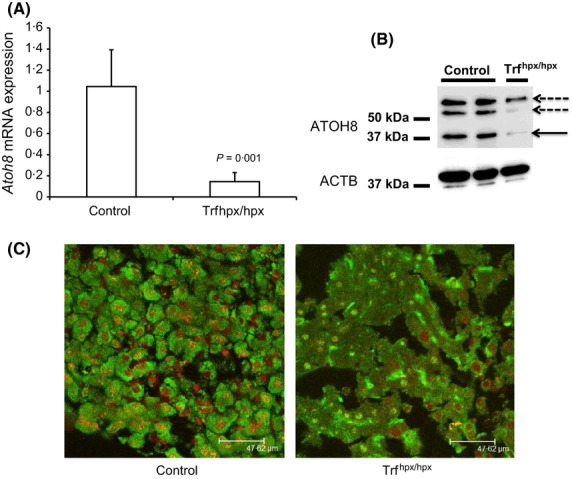
Expression of *Atoh8* in HPX mouse liver. (A) Q-PCR shows relative *Atoh8*mRNA levels as (normalized to *Rpl19,* plotted as fold change relative to control) in 10- to 11-week-old male *Trf*^*hpx/hpx*^ mice compared to control (*Trf*^*hpx/+*^*)* mice (*P* = 0·001 Student's ‘*t*’ test, *n* = 3 for each group). (B) Western blots for ATOH8 protein in liver extracts from two control (*Trf*^*hpx/+*^*)* and one *Trf*^*hpx/hpx*^ mouse. Solid arrow indicates predicted molecular weight of ATOH8 (∽37 kDa), dashed arrows indicate possible homo- or heterodimers. (C) ATOH8 immunostaining (visualized in green) in liver sections from male 7-week-old *Trf*^*hpx/hpx*^ compared to an age- and sex-matched control (*Trf*^*hpx/+*^); counterstain is propidium iodide (red).

We confirmed the significant down regulation of *Atoh8* in liver of *Tfr*^hpx/hpx^ mice by qPCR (*P* = 0·001, Fig3A). In addition, reduced ATOH8 protein was evident by both Western blotting and immunohistochemistry (Fig3B,C) in *Tfr*^hpx/hpx^ mice compared to controls. Thus it appears that ATOH8 upregulation by iron was overridden in *Tfr*^hpx/hpx^ mice in a similar fashion to regulation of liver *Hamp1* levels.

**Figure 4:**
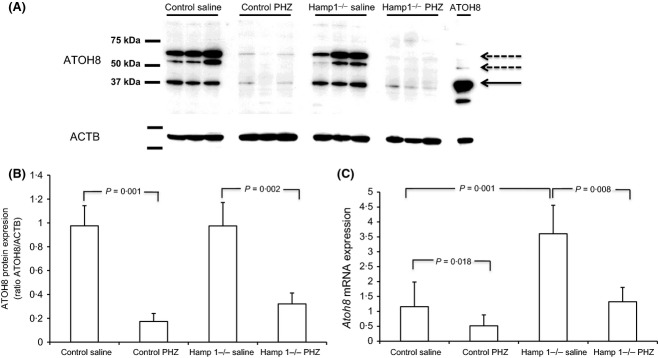
Liver ATOH8 levels in control and Hamp1^−/−^ mice with induced haemolytic anaemia. (A) Western blot showing ATOH8 protein levels in liver extracts from individual mice (three per group). Last lane shows extract from HEK293 cells transfected with *ATOH8* plasmid as a positive control. Solid arrow indicates predicted molecular weight of ATOH8 (∽37 kDa), dashed arrows indicate possible homo- or heterodimers, lower panel shows same blot re-probed for ACTB (β-actin). (B) Densitometry of Western blots performed in panel A showing ratio of ATOH8 to ACTB. (C) qPCR shows *Atoh8*mRNA levels in saline-injected control mice (*n* = 7) *versus* control mice injected with PHZ (*n* = 8) and saline-injected Hamp1^−/−^ mice (*n* = 6) *versus* Hamp1^−/−^ injected with PHZ (*n* = 7). Control mice were wild type littermates. (*Atoh8* levels were normalized to *Rpl19* and plotted as fold change relative to control). Values are means ± SD. Statistical comparisons were made using 1-way anova with Tukey's *post hoc* test.

We reasoned that the reduction in ATOH8 in *Tfr*^hpx/hpx^ mouse liver may be driven by increased erythroid activity. Treatment of rats or mice with PHZ leads to increased erythropoietic rate and suppression of *Hamp1* usually after a lag period of 3 d (Frazer *et al*, 2004; Latunde-Dada *et al*, 2006; Masaratana *et al*, 2012). In mice injected with PHZ there was an approximate 27-fold increase in the percentage of blood reticulocytes (Raja *et al*, 1989), reduced or absent serum iron and a 2–3 fold increase in liver non-haem iron. In normal mice (C57BL/6/129 mixed background) sacrificed 3 d after PHZ treatment, ATOH8 protein levels decreased by around eightfold (*P* = 0·001, Fig4A,B) while *Atoh8* mRNA levels were reduced by around twofold (*P* > 0·018) (Fig4C). We considered the possibility that ATOH8 may be regulated downstream of *Hamp1*, however similar reductions in ATOH8 protein and mRNA levels were observed in *Hamp1*^−/−^ mice treated with PHZ (Fig4A,B,C), suggesting that regulation of ATOH8 is upstream of *Hamp1*. The response of liver ATOH8 protein and mRNA levels to PHZ was also similar in CD1 and C57BL/6 mice and in both male and female mice (data not shown). *Smad7* and *Id1* levels were suppressed by PHZ treatment in control and *Hamp1*^−/−^ mice, however the decrease was only statistically significant in the case of *Smad7* (Fig S1). Thus in another model with increased erythropoiesis and liver iron loading, hepatic ATOH8 levels followed the same downward direction as *Hamp1*.

**Figure 5:**
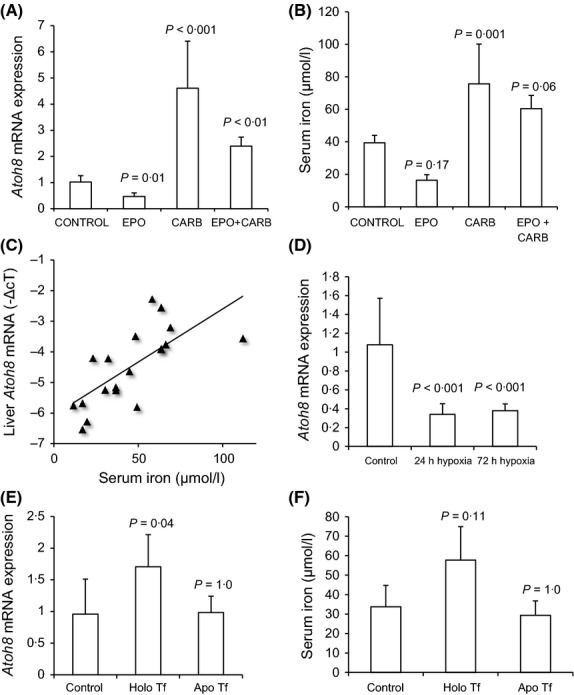
Effect of Hypoxia, EPO, Carboplatin and Transferrin on liver *Atoh8 mRNA* levels. (A) qPCR shows relative *Atoh8* levels (normalized to *Rpl19*; plotted fold-change relative to control). Control (saline-injected; *n* = 7), EPO (*n* = 4), carboplatin (CARB; *n* = 4) and EPO and Carboplatin injected mice (EPO + CARB; *n* = 4). (B) serum iron levels in treated mice. (C) Correlation between serum iron and *Atoh8*mRNA in all mice (Pearson correlation coefficient, *R*^2^ = 0·70, *P* = 0·013, *n* = 19). (D) qPCR of liver *Atoh8*mRNA levels in control (normoxia) and in mice exposed to hypoxia for 24 and 72 h (*Atoh8* levels normalized to *Rpl19*, plotted as fold-change relative to control) (E) *Atoh8* levels in control (saline injected) (*n* = 7), Holo-transferrin (*n* = 4) or Apo-transferrin (*n* = 4) (*Atoh8* levels normalized to *Rpl19*; plotted as fold-change relative to control) (F) serum iron levels in treated mice. Data are presented as mean ± SD. Statistical comparisons were made using 1-way anova with Tukey's *post hoc* test.

Given that PHZ injection results in other effects, such as release of haem and increased oxidative stress, we investigated the effects of other more physiological modulators of erythropoiesis, such as exposure of mice to hypoxia and EPO injection on liver *Atoh8* levels. In mice, EPO injection and exposure to hypoxia lead to increased erythropoiesis and suppression of liver *Hamp1* mRNA (Nicolas *et al*, 2002a; Pak *et al*, 2006). On the other hand, injection of the cytotoxic agent carboplatin, which inhibits erythropoiesis, results in increased liver *Hamp1* (Pak *et al*, 2006; Bartnikas *et al*, 2010). As previously shown, we found that liver *Hamp1* mRNA was suppressed by EPO treatment and increased by carboplatin treatment (Fig S2A). EPO treatment significantly reduced liver *Atoh8* mRNA levels by around twofold compared to control mice (*P* = 0·01), whereas mice treated with carboplatin alone or EPO and carboplatin had significantly higher *Atoh8* mRNA levels (*P* < 0·01) compared to control or EPO-treated mice (Fig5A). Mice treated with carboplatin alone showed the highest induction of *Atoh8* mRNA levels (approximately fourfold induction, *P* < 0·001).

Serum iron levels generally tracked liver *Atoh8* levels (Fig5B) although some of the treatments did not reach statistical significance (EPO treatment and EPO with carboplatin). There was a significant correlation between serum iron and *Atoh8* mRNA levels when all experimental groups were taken together (*R*^2^ = 0·70, *P* = 0·013, *n* = 19, Fig5C). Liver non-haem iron concentration was not significantly affected by EPO or carboplatin treatments alone but increased significantly with carboplatin and EPO treatment (*P* < 0·001, Fig S3) and there was no correlation between liver iron concentration and *Atoh8* mRNA levels (*R*^2^ = 0·41, *P* = 0·07, *n* = 15). Liver *Id1* and *Smad7* mRNA levels were not affected by EPO treatment although levels of both were increased by carboplatin treatment (Fig S4). In contrast to *Atoh8* and *Hamp1*,*Id1* and *Smad7* levels were increased further by carboplatin plus EPO treatments (Fig S4).

Hypoxia is a well-known physiological stimulator of erythropoiesis that is known to suppress liver *HAMP* levels in both mice and man (Nicolas *et al*, 2002a; Talbot *et al*, 2012). Exposure of mice to 24 or 72 h hypoxia reduced liver *Atoh8* mRNA levels by around threefold (*P* < 0·001, Fig5D). Thus, *Atoh8* was regulated by altered erythropoietic activity in the same direction as *Hamp1* and hepatic *Atoh8* mRNA levels correlated with serum iron.

### Holo-transferrin has a direct effect on liver *Atoh8* mRNA levels

Holo-transferrin is thought to be a key regulator of liver *Hamp1*, therefore we investigated whether diferric transferrin had any direct effect on liver *Atoh8* mRNA levels. C57BL/6 mice were treated with 10 mg of holo-transferrin via i.p injection and sacrificed 6 h later, a treatment previously shown to increase liver *Hamp1* levels without changing liver iron (Ramos *et al*, 2011). As previously shown (Ramos *et al*, 2011) liver *Hamp1* mRNA levels were significantly induced by holo-transferrin but not apo-transferrin (Fig S2B). Treatment of mice with holo-transferrin resulted in an approximate twofold increase in *Atoh8* mRNA levels (*P* = 0·037) whereas apo-transferrin had no effect (Fig5E). Serum iron increased in holo-transferrin injected mice although this did not reach statistical significance (*P* = 0·11, Fig5F). There was no significant effect of holo- or apo-transferrin on liver non-haem iron levels (Fig S3) or on hepatic *Smad7* and *Id1* mRNA levels (Fig S4A,B). Thus serum levels of diferric transferrin can directly regulate liver *Atoh8* mRNA levels.

## Discussion

This study establishes ATOH8 as a novel candidate transcriptional regulator of hepatic *HAMP* levels and cellular pSMAD1,5,8 levels. ATOH8 stimulated *HAMP* transcription while mutation of E-boxes within the *HAMP* promoter attenuated ATOH8-dependent *HAMP* transcriptional responses and CHIP assays provided additional evidence that ATOH8 binds to these E-boxes both *in vivo and in vitro*. Although MYC, MAX, USF1 and 2 can bind the same E-boxes *in vitro* (Bayele *et al*, 2006), the physiological role of these proteins in iron metabolism *in vivo* remains unclear. In the case of USF1 and 2 with the well-known exception of a *Usf2* knockout mouse in which the *Hamp1* locus was also disrupted (Nicolas *et al*, 2001), selective knock out of either *Usf1* (Nicolas *et al*, 2001) or *Usf2* (Nicolas *et al*, 2002b) in mice has no effect on liver *Hamp1* levels or iron metabolism. Furthermore, analysis of the published array data supplied by Kautz *et al* (2008) for iron loaded and iron deficient mouse liver (Data available at the National Center for Biotechnology Information [NCBI] Geo database (Edgar *et al*, 2002), accession GSE10421) in C57 and DBA strains shows that liver *Usf1* mRNA was not iron-regulated in either strain whereas *Usf2* mRNA was decreased by iron loading in both strains of mice but also decreased in iron deficiency. In the HPX mouse liver, we found no change in *Usf1* and an increase in *Usf2* mRNA levels (data not shown). In contrast, hepatic *Atoh8* and *Hamp1* mRNA levels correlated *in vivo* in mice over a wide range of conditions of altered iron metabolism (iron overload, iron deficiency, hypotransferrinaemia, hypoxia, PHZ, EPO and carboplatin treatment). Moreover, downregulation of ATOH8 by increased erythropoietic drive occurred in *Hamp1* null mice, a scenario consistent with ATOH8 being an upstream regulator of *Hamp1*. *In vivo*, there is likely to be competition between the various E-box proteins for binding to the *HAMP* promoter and which protein binds would depend on hepatic expression levels, DNA binding affinity as well as other tissue and gene-specific factors. At present it is unclear what the nature of the higher molecular weight bands found on ATOH8 liver Western blots (Figs3B and 4A) are, however they appear to be regulated in the same manner as the 37 kDa ATOH8 band. bHLH proteins, such as MYC and MAX homo or heterodimerize with each other in order to bind DNA (Blackwell *et al*, 1993). It is possible that these higher molecular weight bands are SDS-resistant dimers with other bHLH proteins although further work will be required to identify these.

ATOH8 also regulated pSMAD1,5,8 levels, providing an additional mechanism by which ATOH8 could influence *HAMP* levels. This was supported by the finding that mutation of the BMP-RE in the *HAMP* promoter also attenuated ATOH8-dependent *HAMP* transcription. It has been suggested that increased erythropoietic activity in mice after PHZ treatment can attenuate BMP6 signalling and decrease liver *Hamp1* levels without any change in pSMAD1,5,8 levels (Frazer *et al*, 2012). We speculate that the reduction in liver ATOH8 as observed following acute PHZ treatment could negate the effect of increased BMP6 levels on pSMAD1,5,8, levels and reduce E-box-dependent transcriptional activation of *HAMP*.

Previous work has established that hypoxia and EPO suppresses *HAMP* indirectly through stimulation of erythropoiesis while inhibition of erythropoiesis with carboplatin leads to increases in *HAMP* (Pak *et al*, 2006; Talbot *et al*, 2012). Liver *Atoh8* levels responded to these stimuli in a similar manner and direction to *HAMP*, suggesting *Atoh8* responds to the same systemic cues as *HAMP*. What these cues are remains to be fully elucidated.

**Figure 6:**
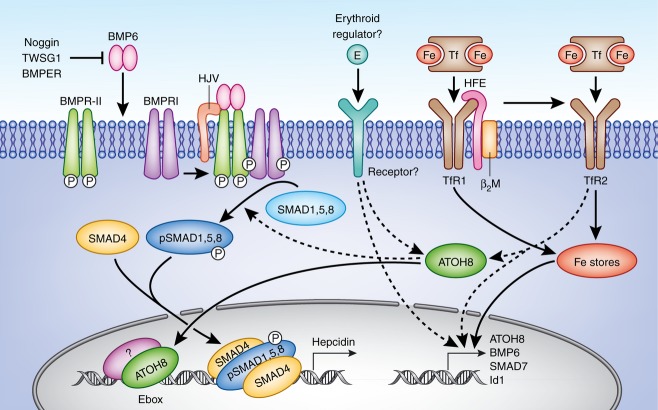
Working model Hepatic BMP6, ATOH8, SMAD7 and ID1 levels are increased by increased tissue iron stores, however only ATOH8 levels are decreased by increased erythropoietic activity. ATOH8 regulates *HAMP* levels via direct transcriptional activation of the *HAMP* gene via binding to E-boxes (possibly as a heterodimer with other as yet unidentified bHLH or bHLH-ZIP proteins) and by modulation of cellular pSMAD1,5,8 levels. Reductions in hepatic ATOH8 levels under increased erythroid activity lead to reduced pSMAD1,5,8 levels and E-box dependent *HAMP* transcription. Dashed arrows indicate hypothetical connections between ATOH8 and other proteins. ATOH8 may be a component of signal transduction pathways linking *HAMP* transcription with levels of diferric transferrin (via iron-sensing molecules such as HFE and TfR2) and with erythroid activity (by as yet unidentified erythroid regulators).

Transferrin saturation correlates directly with erythropoietic activity (Frazer *et al*, 2004) while numerous studies *in vivo* show that serum diferric transferrin levels correlate with liver *Hamp1* levels in mice (Wilkins *et al*, 2006; Bartnikas *et al*, 2010; Li *et al*, 2010; Ramos *et al*, 2011). It is thought that increased diferric transferrin levels leads to stabilization of TfR2, possibly due to binding of Hfe (Robb & Wessling-Resnick, 2004; Schmidt *et al*, 2008), generating an as yet unidentified signal leading to increased *HAMP* levels. Our data, showing that that holo-transferrin also directly regulates liver *Atoh8* levels, suggest that this signalling pathway may involve ATOH8 (Fig6). However ATOH8 levels were also suppressed in *Hamp1*^−/−^ mice after PHZ treatment where plasma iron remains high in the former (Masaratana *et al*, 2012) Thus it is possible that other as yet unidentified erythroid factor (s) released from rapidly developing erythrocytes or the bone marrow also regulate *Atoh8* levels (Fig6). Interestingly, hepatic *Atoh8* levels were not increased in Hfe knockout mice, in contrast to other iron loaded models (Kautz *et al*, 2008, 2009). This indicates that HFE may also be required for regulation of ATOH8. Further work is required to uncover the link between iron sensing molecules and ATOH8.

To date, none of the other factors known to be involved in BMP signalling, including BMPs2, 4 and 9, Alk2 (ACVR1), Alk3 (BMPR1A), Hjv (HFE2) and TMPRSS6, have been shown to be iron-regulated or regulated by changes in erythropoietic activity *in vivo*. SMAD7 is a known inhibitor of the BMP signalling pathway while ID1 is an HLH transcription factor of unknown function, which acts as a dominant negative inhibitor of other bHLH proteins (Pesce & Benezra, 1993) because it lacks a basic DNA binding domain but can still form heterodimers (Langlands *et al*, 1997; Bounpheng *et al*, 1999). SMAD7 and ID1 are iron regulated (Kautz *et al*, 2008) and therefore could influence *HAMP* transcription under increased erythropoietic activity. However, given that levels of both *Smad7* and *Id1* mRNA decreased in livers of mice treated with PHZ, and were unaffected by holo-transferrin or EPO treatments, its seems unlikely that either are of major importance in *HAMP* suppression under enhanced erythropoietic drive.

BMP6 has been dubbed the iron stores regulator as several studies have suggested that tissue iron rather than serum iron is the dominant regulatory factor for BMP6 (Ramos *et al*, 2011; Frazer *et al*, 2012). Recently, it has been revealed that liver *Hamp1* levels increased markedly in *Bmp6* knockout mice following chronic iron loading, indicating other pathways in addition to BMP6 are involved in the regulation of *Hamp1* by iron (Ramos *et al*, 2011). A plausible explanation, based on our data, is that the effects of iron on *Hamp1* expression in the absence of BMP6 are mediated by stimulation of the Atoh8 pathway. Thus BMP6 may modulate *Hamp1* in response to changes tissue iron whereas Atoh8 may regulate responses to serum iron and/or changes in erythropoietic activity that dominate under certain circumstances. Given that ATOH8 affects at least two pathways which regulate *HAMP* transcription (BMP signalling and E-Box dependent transcription) ATOH8 could have a strong influence hepatic *Hamp1*. This would allow *Hamp1* responses to various stimuli, explaining how suppression occurs by increased erythropoietic activity even in the face of liver iron loading and increased BMP6 levels.

In summary, we identify ATOH8 as a novel transcriptional regulator of *HAMP* via two independent mechanisms: E-box dependent transcriptional activation; and regulation of cellular pSMAD1,5,8 levels. The regulation of liver ATOH8 levels observed in mice with altered erythropoiesis suggests ATOH8 as a novel physiological regulator of *HAMP*. ATOH8 may link erythropoietic activity and iron-sensing molecules to *HAMP* transcription and will open up new avenues of research leading to improved therapies and management of iron overload disorders, such as haemochromatosis and β-thalassaemia.

## Supporting Information

Fig S1. Hepatic *Id1* and *Smad7* mRNA levels in control and Hamp1^−/−^ mice with induced haemolytic anemia. **Fig S2.** Effect of modulators of erythropoiesis and changes in transferrin levels on hepatic *Hamp1* mRNA expression. Fig S3. Effect of modulators of erythropoiesis and changes in transferrin levels on liver non-heme iron levels. Fig S4. Effect of modulators of erythropoiesis and changes in transferrin levels on hepatic *Id1* and *Smad7* expression. Table SI. Primer sequences.
