# Supplementary material for: The transcription factor ATOH8 is regulated by erythropoietic activity and regulates HAMP transcription and cellular pSMAD1,5,8 levels
Source: Br J Haematol. 2013 Nov 16;164(4):586–96. doi: 10.1111/bjh.12649 (PMC4232863; doi:10.1111/bjh.12649)
Supplement: Supplementary file 1 — Fig S1. Hepatic Id1 and Smad7 mRNA levels in control and Hamp1−/− mice with induced haemolytic anemia. Fig S2. Effect of modulators of erythropoiesis and changes in transferrin levels on hepatic Hamp1 mRNA expression. Fig S3. Effect of modulators of erythropoiesis and changes in transferrin levels on liver non-heme iron levels. Fig S4. Effect of modulators of erythropoiesis and changes in transferrin levels on hepatic Id1 and Smad7 expression. Table SI. Primer sequences. [file bjh0164-0586-SD1.doc]

Supplementary Table 1. Mean (standard deviation) changes from baseline in Investigator’s Global Assessment (IGA) score and symptom scores at weeks 8 and 12, showing statistical significance for comparisons between liarozole and placebo groups (intent-to-treat population).

|  | **Placebo group**  **(*n* = 9)** | **75 mg liarozole**  **(*n* = 27)** | **150 mg liarozole**  **(*n* = 28)** |
| --- | --- | --- | --- |
| **Change from baseline in IGA score** | | | |
| Week 8 | –0.1 (0.8) | –1.3 (0.7); *P*=0.003 | –1.5 (0.9); *P*=0.004 |
| Week 12 | –0.2 (1.0) | –1.4 (0.8); *P*=0.017 | –1.5 (1.0); *P*=0.010 |
| **Change from baseline in symptom scores** | | | |
| Scaling |  |  |  |
| Week 8 | –3.1 (6.7) | –12.0 (8.1); *P*=0.016 | –13.1 (8.3); *P*=0.017 |
| Week 12 | –2.7 (7.3) | –11.7 (7.4); *P*=0.050 | –13.5 (9.0); *P*=0.010 |
| Erythema |  |  |  |
| Week 8 | –3.0 (5.3) | –5.9 (6.5)^a^ | –5.0 (7.9); *P*=0.76 |
| Week 12 | –2.2 (3.4) | –5.1 (6.1)^a^ | –5.6 (7.7); *P*=0.65 |
| Pruritus |  |  |  |
| Week 8 | –4.3 (8.4) | –10.0 (10.5)^a^ | –7.0 (9.4); *P*=0.76 |
| Week 12 | –5.7 (9.5) | –9.4 (10.7)^a^ | –6.3 (9.8); *P*=0.76 |

These data were generated *post hoc*, and have been included at the request of journal peer reviewers. Last observation carried forward; *P* values for comparison with placebo using a two-sided Wilcoxon-Mann-Whitney rank sum test, adjusted for multiplicity of the eight secondary variables with the Simes-Hommel intersection-union procedure. ^a^Statistical analysis not considered because the comparison between the 150 mg liarozole group and placebo group was not significant.

Supplementary Table 2. Liarozole pre-dose and near-peak plasma concentrations

| **Liarozole plasma concentration, ng/mL** | **75 mg liarozole** | **150 mg liarozole** |
| --- | --- | --- |
| Week 4, mean (SD)  Pre-dose concentration  Near-peak concentration | 32.0 (31.5)^a^  906 (282)^c^ | 43.2 (32.5)^b^  2070 (606)^b^ |
| Week 8, mean (SD)  Pre-dose concentration | 34.4 (42.5)^a^ | 40.4 (31.8)^c^ |
| Week 12, mean (SD)  Pre-dose concentration  Near-peak concentration | 30.2 (33.7)^d^  910 (395)^d^ | 51.0 (41.9)^c^  2176 (760)^b^ |

SD, standard deviation. ^a^*n* = 24; ^b^*n* = 27; ^c^*n* = 25; ^d^*n* = 26.

Supplementary Table 3. Summary of adverse events (safety population)

|  | **Placebo group  (*n* = 9)** | **75 mg liarozole group  (*n* = 27 )** | **150 mg liarozole group  (*n* = 28)** | **Combined liarozole groups  (*n* = 55)** |
| --- | --- | --- | --- | --- |
| Number of patients reporting AEs, *n* (%) | 6 (67) | 19 (70) | 20 (71) | 39 (71) |
| Number of AEs, *n* | 11 | 91 | 76 | 167 |
| Number of patients reporting treatment-related AEs, *n* (%) | 2 (22) | 16 (59) | 17 (61) | 33 (60) |
| Number of treatment-related AEs, *n* | 4 | 67 | 51 | 118 |
| Number of patients who discontinued treatment, *n* (%) |  |  |  |  |
| Temporary discontinuation | 0 | 2 (7)^a^ | 2 (7)^b^ | 4 (7) |
| Permanent discontinuation | 0 | 0 | 1 (4)^c^ | 1 (2) |

AE, adverse event. ^a^One incidence each of moderate pruritic rash (possibly related to study drug) and moderate constipation; ^b^moderate rash and moderate nausea, both possibly or probably related to study drug; ^c^patient had two AEs (moderate rash and moderate dry skin), possibly related to study drug.

Supplementary Figure 1. Change in mean Dermatology Life Quality Index (DLQI) over time (intent-to-treat population; last observation carried forward)

**P*=0.014 (vs. placebo), ^a^*n* = 27 in 150 mg liarozole group at week 4.
